# A Cross‐Cohort Study Examining the Associations of Metabolomic Profile and Subclinical Atherosclerosis in Children and Their Parents: The Child Health CheckPoint Study and Avon Longitudinal Study of Parents and Children

**DOI:** 10.1161/JAHA.118.011852

**Published:** 2019-07-09

**Authors:** Markus Juonala, Susan Ellul, Debbie A. Lawlor, Diana L. Santos Ferreira, John B. Carlin, Michael Cheung, Terence Dwyer, Melissa Wake, Richard Saffery, David P. Burgner

**Affiliations:** ^1^ Department of Medicine University of Turku Finland; ^2^ Division of Medicine Turku University Hospital Turku Finland; ^3^ Murdoch Children's Research Institute Parkville Victoria Australia; ^4^ The Medical Research Council Integrative Epidemiology Unit at the University of Bristol Bristol United Kingdom; ^5^ National Institute for Health Research Bristol Biomedical Research Centre Bristol United Kingdom; ^6^ Population Health Science Bristol Medical School University of Bristol United Kingdom; ^7^ Royal Children's Hospital Parkville Victoria Australia; ^8^ The George Institute for Global Health University of Oxford United Kingdom; ^9^ Department of Pediatrics University of Melbourne Parkville Victoria Australia; ^10^ Department of Pediatrics Monash University Clayton Victoria Australia

**Keywords:** intima‐media thickness, pediatric, pulse wave velocity, Epidemiology

## Abstract

**Background:**

High‐throughput nuclear magnetic resonance profiling of circulating metabolites is suggested as an adjunct for cardiovascular risk evaluation. The relationship between metabolites and subclinical atherosclerosis remains unclear, particularly among children. Therefore, we examined the associations of metabolites with carotid intima‐media thickness (cIMT) and arterial pulse wave velocity (PWV).

**Methods and Results:**

Data from two independent population‐based studies was examined; (1) cross‐sectional associations with cIMT and PWV in 1178 children (age 11–12 years, 51% female) and 1316 parents (mean age 45 years, 87% female) from the CheckPoint study (Australia); and (2) longitudinal associations in 4249 children (metabolites at 7–8 years, PWV at 10–11 years, 52% female), and cross‐sectional associations in 4171 of their mothers (mean age 48 years, cIMT data) from ALSPAC (The Avon Longitudinal Study of Parents and Children; UK). Metabolites were measured by the same nuclear magnetic resonance platform in both studies, comprising of 69 biomarkers. Biophysical assessments included body mass index, blood pressure, cIMT and PWV. In linear regression analyses adjusted for age, sex, body mass index, and blood pressure, there was no evidence of metabolite associations in either children or adults for cIMT at a 10% false discovery threshold. In CheckPoint adults, glucose was positively, and some high‐density lipoprotein‐cholesterol derived measures and amino acids (glutamine, histidine, tyrosine) inversely associated with PWV.

**Conclusions:**

These data suggest that in children circulating metabolites have no consistent association with cIMT and PWV once adjusted for body mass index and blood pressure. In their middle‐aged parents, some evidence of metabolite associations with PWV were identified that warrant further investigation.

**Clinical Perspective**

**What Is New?**

- The analyses of two large independent population‐based studies (CheckPoint, ALSPAC [The Avon Longitudinal Study of Parents and Children]) revealed limited evidence of an association between nuclear magnetic resonance–based metabolomic profile and subclinical phenotypes of atherosclerosis in children following adjustment for age and sex.
- These associations were generally attenuated toward the null with further adjustment for body mass index and systolic blood pressure.
- In the CheckPoint adults, but not CheckPoint or ALSPAC children, glucose was positively and some high‐density lipoprotein‐cholesterol derived measures and amino acids (glutamine, histidine and tyrosine) were inversely associated with pulse wave velocity independently of body mass index and systolic blood pressure.

**What Are the Clinical Implications?**

- Our findings from general populations do not support introduction of nuclear magnetic resonance metabolic profiles into pediatric cardiovascular practice.
- Among adults we observed some signals related to preclinical atherosclerosis, but the most robust evidence to date with this nuclear magnetic resonance platform comes from prospective analyses with disease endpoints and replication across three independent cohorts.

## Introduction

Atherosclerotic cardiovascular disease (CVD) is the leading cause of mortality worldwide. Although its clinical complications usually become evident only from midlife, atherosclerosis has its origins in early life.^1^ Traditional risk factors in childhood, including dyslipidemia, elevated blood pressure (BP), tobacco smoke exposure, diabetes mellitus, and obesity, are associated with vascular phenotypes indicative of subclinical atherosclerosis and subsequent risk of CVD.^2^, ^3^, ^4^, ^5^ However, the first clinical CVD events often occur among adults without traditional risk factors who would be classified as being at low or intermediate risk based on widely used CVD risk algorithms.^6^

There has been increasing interest in the potential of the metabolic profile (metabolomics) to reveal both novel insights into pathophysiology and contribute to improved clinical prediction of atherosclerotic CVD.^7^, ^8^ Nuclear magnetic resonance (NMR) methodology enables the rapid, inexpensive, and reproducible quantification of circulating lipids and metabolites, including several amino acids, glycolysis‐related metabolites, and ketone bodies.^9^ In adults, distinct metabolomic profiles have been shown to predict all‐cause mortality, cardiovascular events, and type 2 diabetes mellitus.^7^, ^8^, ^10^ However, there are a paucity of equivalent data for early subclinical markers of atherosclerosis, especially in childhood.

In the present study, we first investigated the age‐ and sex‐adjusted associations of NMR metabolomic profile with measures of subclinical atherosclerosis, carotid intima‐media thickness (cIMT), and arterial pulse‐wave velocity (PWV) in children and their mothers. We then investigated these associations with additional adjustment for body mass index (BMI) and systolic BP, to determine whether the NMR metabolomic profile has the potential to improve risk prediction over and above these established risk factors that are readily accessible to both clinical and public health settings. These vascular measures are widely used in epidemiological studies, given that they are associated with cardiovascular risk factors and independently predict cardiovascular events in adults.^4^, ^5^

## Methods

We utilized data from the Child Health CheckPoint study (“CheckPoint”) and ALSPAC (The Avon Longitudinal Study of Parents and Children).

**Figure 1:**
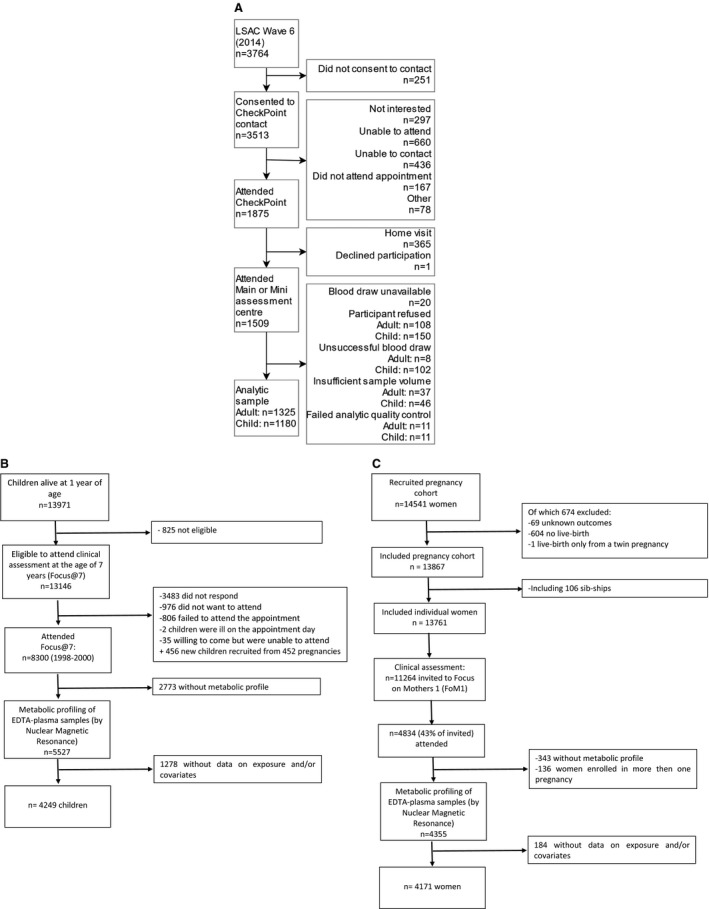
Flow charts of CheckPoint and ALSPAC (The Avon Longitudinal Study of Parents and Children) studies. A, CheckPoint, (B) ALSPAC children, and (C) ALSPAC adults. LSAC indicates Longitudinal Study of Australian Children.

CheckPoint was a detailed cross‐sectional assessment of physical health and biomarkers in a population‐based national sample of Australian children (aged 11–12 years) and 1 of their parents, conducted between February 2015 and March 2016.^11^, ^12^ CheckPoint is a substudy nested between waves 6 (2014) and 7 (2016) of data collection in the Longitudinal Study of Australian Children (LSAC), which recruited a nationally representative sample of 5107 (57.2% uptake) 0‐ to 1‐year‐old infants in 2004 and has since followed them biennially.^13^ The CheckPoint study population included here consisted of 1178 children (mean age, 12.0 years; 51.0% females) and 1316 adults (mean age, 44.6 years; 86.6% females, predominantly the child's mother) with metabolomics, cIMT, and/or PWV measurements available (Figure 1). The protocol was approved by The Royal Children's Hospital (Melbourne, Australia) Human Research Ethics Committee and the Australian Institute of Family Studies Ethics Committee. The attending parent/caregiver gave written informed consent for themselves and their child, and optional consent for the collection and analysis of biological samples.

ALSPAC is a prospective, population‐based birth cohort study that recruited 14 541 pregnant women resident in the South West of England with expected dates of delivery between April 1, 1991 and December 31, 1992. These women, their partners, and their offspring have been followed up for 28 years with repeat questionnaires, hands‐on clinic assessments, and record‐linkage, with full details of the study published elsewhere.^14^, ^15^ For the current study, we used data from 4249 offspring who had metabolites measured at age 7 to 8 years and arterial PWV measured at age 11 to 12 years, together with 4171 mothers who had metabolites and cIMT measured cross‐sectionally at mean age 48 years (Figure 1). These assessment time points were selected to be as close as possible to those of the CheckPoint children and parents. Ethical approval for the study was obtained from the ALSPAC Ethics and Law Committee and from the UK NHS National Health Service Local Research Ethics Committees. Participants have provided informed consent for the use of the data, with the main caregiver (most commonly their mother) providing consent for their child and the child providing assent to any specific data collection or blood sampling.

### Metabolomics

In CheckPoint, semifasted (median, 4.2 hours postprandial) peripheral blood was collected and processed within 4 hours at an on‐site processing laboratory, with serum aliquots frozen at −80°C for batch analysis.^12^, ^16^ In the ALSPAC, nonfasted peripheral blood was collected in children and fasted (overnight or minimum 6 hours) in mothers; in both, samples were processed within 4 hours and stored at −80°C.^17^, ^18^ Metabolomic profiling^9^, ^16^ was done using the Nightingale NMR metabolomics platform (Helsinki, Finland), and 228 metabolic traits (or their ratios) were quantified in serum (CheckPoint) or EDTA‐plasma (ALSPAC). With this platform, the coefficients of variation for the metabolomics variables in the present study vary between 1.5% and 12.5%, with very large very‐low‐density lipoprotein (12.5%), very large high‐density lipoprotein (HDL; 10.6%), and citrate (10.1%) being the only variables having coefficients of variation over 10%.^19^ In present cohorts for most variables, data were available in >99% of participants. Many of these metabolomics measures correlate substantially both in adults and children.^16^, ^20^ For clarity and in line with earlier research with this platform,^21^ we therefore focused on 69 lipids and metabolites in this study. We excluded the 5 ratio measures for each of the 14 lipoprotein subclass particles. In addition, the 7 other measures within each of the lipoproteins (esterified cholesterol, free cholesterol, total cholesterol, triglycerides, phospholipids, total lipids, and particle concentration) are derived from other metabolites and all highly correlated. Therefore, we only reported total lipids for each of the lipoprotein subclass particles. Glycoprotein acetyl was excluded given that it has been analyzed in another report. We excluded glycerol, glycine, and pyruvate because they were only quantified for CheckPoint and not the ALSPAC because of differences in the sample types (plasma versus serum) analyzed.

### Age, Sex, BMI, BP, and Socioeconomic Status

In CheckPoint, children's date of birth and sex were from LSAC records. In adults, self‐reported sex and date of birth were acquired at time of assessment. Trained assessors measured participant's height using a Leicester height meter, without shoes and in light clothing, to the nearest 0.1 cm, in duplicate. A third measurement was taken if the difference of the first 2 measurements was >0.5 cm. The mean of all measurements was used in analyses. An InBody230 bioelectrical impedance analysis scale (Biospace Co. Ltd., Seoul, South Korea) measured weight to the nearest 0.1 kg. Body mass index (BMI) was calculated by dividing the weight (kg) by the height squared (m^2^). Supine brachial artery BP was measured after a 7‐minute rest up to 3 times using the SphygmoCor XCEL (AtCor Medical Pty Ltd., West Ryde, NSW, Australia), and the mean values were used in analysis. Parental education data were obtained from questionnaires and categorized into 3 groups: tertiary educated, completed high school (year 12) only, or completed less than year 12.

In ALSPAC children, sex and birthdate were recorded in the delivery room and abstracted from obstetric records and/or birth notifications. Exact age was determined from the child's date of birth and date of the examination. Weight (to the nearest 0.1 kg), using seca scales (seca, Hamburg, Germany), and height (to the nearest 0.1 cm), using a Leicester height meter, were both measured with the child unshod and in light clothing at age 7 years (at the time of metabolite measurement). BMI was calculated as in CheckPoint. Seated BP was also measured at age 7 years with the child at rest, their arm supported, and the correct cuff size (after measurement of arm circumference), using an Omron M6 upper arm monitor (Omron Healthcare, Inc., Lake Forest, IL). In ALSPAC mothers at mean age 48 years, weight was measured to the nearest 0.1 kg using Tanita scales (Tanita Corporation, Tokyo, Japan); height was measured to the nearest 0.1 cm using a Harpenden stadiometer (women were unshod and in light clothing). Seated BP was measured with the woman at rest, her arm supported and the correct cuff size (after measurement of arm circumference), using an Omron M6 upper arm monitor (Omron Healthcare, Inc.,). The mean of 2 BP measurements were used in children and adults. Maternal education was obtained from questionnaires and categorized into 3 groups: university degree or above, A‐level (Advanced Level; exams taken in different subjects usually at age 18 years), or below A‐level.

### Vascular Phenotypes

#### Carotid intima‐media thickness

In CheckPoint, cIMT was measured as previously described.^22^ B‐mode ultrasound carotid artery images were acquired using standardized protocols. cIMT was measured ≈10 mm proximal to the carotid bulb, over a distance of 5 to 10 mm. Participants lay supine with their head turned 45 degrees to the left to expose the right side of the neck. A 10‐MHz linear array probe (Vivid‐I; GE Healthcare, Chicago, IL) obtained cine loops of the right common carotid artery, in triplicate. Modified 3‐lead ECG captured cardiac cycle information concurrently. All images were transferred to digital storage for archiving. Six raters measured cIMT in the ultrasound images using software available in Carotid Analyzer (Medical Imaging Applications, Coralville, IA). The within‐ and between‐observer coefficients of variation were 4.9% and 6.2%, respectively.^22^

In the ALSPAC, mothers’ cIMT measurements were acquired from both the left and right common carotid artery arteries, using high‐resolution B‐ultrasound and scanning longitudinally 1 cm proximal to the carotid bifurcation following a standardized protocol. A Zonare Z One Ultra convertible ultrasound system with an L10‐5 linear transducer was used. Images were focused on the posterior (far) wall of the artery, and the zoom function was used to magnify the area. Ten‐second cine loops were recorded in DICOM format and analyzed offline using Carotid Analyzer for Research (Vascular Research Tools 5; Medical Imaging Applications, LLC, Coralville, IA). Three consecutive cardiac cycles were identified, and 3 measures of cIMT were taken from end‐diastolic frames and averaged. This was done for both right and left carotid arteries. The mean of the left‐ and right‐sided readings was used in all analyses. Images were analyzed by a single trained reader. cIMT was not measured in the offspring during childhood.

### Pulse Wave Velocity

In CheckPoint, carotid‐femoral PWV was determined using the SphygmoCor XCEL (AtCor Medical, Sydney, NSW, Australia).^23^ After a 7‐minute rest, assessors obtained velocity (distance/time) measurements 1 to 3 times while participants lay supine. Further analyses used the mean of the measurements. Simultaneously recorded carotid waveform, using tonometric applanation, and a femoral waveform, using a cuff placed around the upper thigh inflated to subdiastolic pressure, provided the time component of PWV. A tape measure was used to record the distance from the carotid pulse to the suprasternal notch to right femoral pulse (estimated by the crease between thigh and torso when the knee was bent to 90 degrees) to the top of the thigh cuff.

In the ALSPAC, offspring PWV was assessed using applanation tonometry at age 10. Pressure‐pulse waveforms were recorded transcutaneously using a high‐fidelity micromanometer (SPC‐301; Millar Instruments, Houston, TX) from the radial and carotid pulse synchronous with the ECG signal, which provides an R‐timing reference. Integral software processed the data to calculate the mean time difference between R‐wave and pressure wave on a beat‐to‐beat basis over 10 seconds, and the PWV was then calculated using the mean time difference and arterial path length between the 2 recording points (SphygmoCorversion 7.1; ScanMed Medical, Moreton‐in‐Marsh, UK).^24^ PWV was not measured in ALSPAC mothers.

### Statistical Analysis

To describe the study participants, continuous variables were presented as mean (SD) for continuous variables and as number (%) for categorical variables. Because of differences in units and distributions of metabolite measures, metabolite concentrations were scaled to SD units separately for children and adults in each cohort to make comparisons across measures more meaningful. The correlation between parent‐child metabolite levels was estimated using Spearman's correlation coefficients.

Linear regression models were fitted separately for CheckPoint and ALSPAC children and parents with each metabolite concentration as the explanatory variable and cIMT (CheckPoint children and parents, ALSPAC mothers) and PWV (CheckPoint children and parents, ALSPAC children) as the outcomes. Coefficients are presented as difference in mean cIMT (mm) or PWV (m/s) per 1‐SD unit increase in metabolite concentration, with 95% confidence intervals. Associations were initially adjusted for age and sex, known to impact specific metabolite levels, and then additionally for BMI and systolic BP given that these are previously described risk factors associated with cIMT and PWV^2^, ^25^ *P* values were adjusted using the method of Benjamini–Hochberg with a false discovery rate of 10% to account for the number of metabolites examined.^26^

Analysis was performed using Stata (version 15.1; StataCorp LP, College Station, TX) and R software (version 3.5.0; R Foundation for Statistical Computing, Vienna, Austria).

## Results

**Figure 2:**
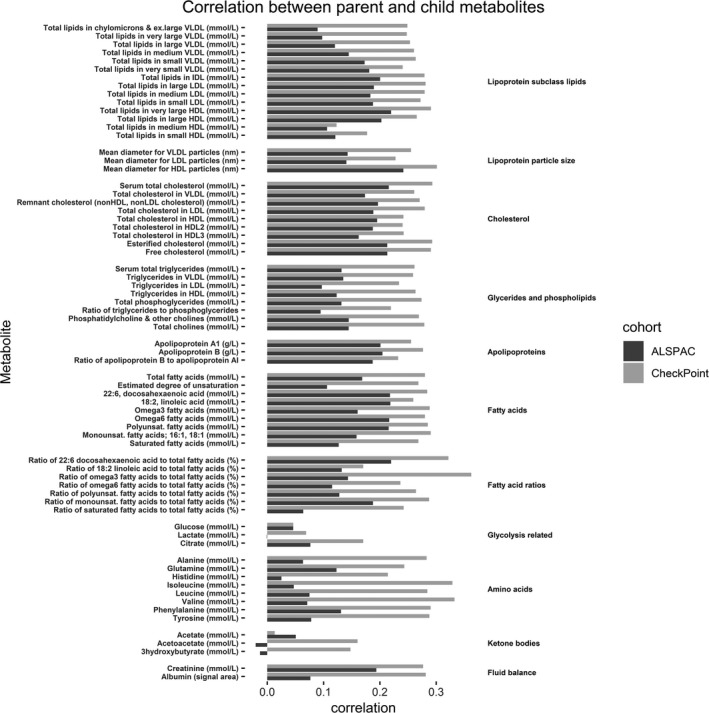
Correlation between parent and child metabolites. Bars represent Spearman's correlation coefficients. HDL indicates high‐density lipoprotein; IDL, intermediate‐density lipoprotein; LDL, low‐density lipoprotein; VLDL, very‐low‐density lipoprotein.

Characteristics of the participants in both cohorts are shown in Table 1 and mean metabolite levels in Table 2. Parent‐child correlations of metabolite levels in both cohorts are shown in Figure 2.

**Table 1:** Characteristics of the Study Cohort

Characteristic	Children		Adults	
	N	Mean (SD)^a^	N	Mean (SD)^a^
CheckPoint (Australia)				
Age, y	1178	12.0 (0.4)	1316	44.6 (5.2)
Male, %	1178	575 (48.8)	1316	177 (13.5)
BMI, kg/m^2^	1177	19.2 (3.3)	1312	27.8 (6.0)
Mean systolic BP, mmHg	1002	108 (8)	1200	121 (13)
Mean cIMT, mm	1170	0.50 (0.06)	1294	0.57 (0.08)
Arterial pulse‐wave velocity, m/s^b^	1141	4.44 (0.55)	1184	6.96 (1.12)
Education, % (< year 12/year 12/tertiary education)			1174	20/43/37
ALSPAC (UK)				
Age at exposure assessment, y	4249	7.5 (0.3)	4171	47.9 (4.4)
Age at outcome assessment, y	4249	10.6 (0.2)	4171	47.9 (4.4)
Male, %	4249	2156 (51)	4171	0 (0)
BMI, kg/m^2^	4249	16.2 (1.9)	4171	26.5 (5.2)
Mean systolic BP, mmHg	4249	98 (9)	4171	118 (12)
Mean cIMT, mm			4171	0.56 (0.06)
Arterial pulse‐wave velocity, m/s^c^	4249	7.53 (1.21)		
Education, % (< A‐level^d^/A‐level/university degree or above)			3883	52/29/19

ALSPAC indicates The Avon Longitudinal Study of Parents and Children; BMI, body mass index; BP, blood pressure; cIMT, carotid intima‐media thickness.

a Mean (SD) for normally distributed variables, n (%) for categorical variables.

b Carotid‐femoral pulse‐wave velocity.

c Carotid‐radial pulse wave velocity.

d Advanced level; exams taken in different subjects usually at age 18years.

**Table 2:** Mean Metabolite Levels in Children and Parents in Study Cohorts

Metabolite	CheckPoint		ALSPAC	
	Children	Parents	Children	Parents (Mothers)
	Mean (SD)	Mean (SD)	Mean (SD)	Mean (SD)
Lipoprotein subclass lipids				
Total lipids in chylomicrons and ex.large VLDL, mmol/L	0.03 (0.03)	0.04 (0.04)	0.03 (0.02)	0.02 (0.02)
Total lipids in very large VLDL, mmol/L	0.06 (0.07)	0.08 (0.10)	0.06 (0.06)	0.04 (0.06)
Total lipids in large VLDL, mmol/L	0.22 (0.21)	0.295 (0.30)	0.19 (0.16)	0.17 (0.20)
Total lipids in medium VLDL, mmol/L	0.45 (0.27)	0.56 (0.40)	0.41 (0.20)	0.41 (0.29)
Total lipids in small VLDL, mmol/L	0.39 (0.15)	0.50 (0.22)	0.53 (0.12)	0.55 (0.21)
Total lipids in very small VLDL, mmol/L	0.33 (0.07)	0.43 (0.11)	0.48 (0.07)	0.54 (0.12)
Total lipids in IDL, mmol/L	0.82 (0.18)	1.00 (0.24)	0.94 (0.18)	1.12 (0.27)
Total lipids in large LDL, mmol/L	0.94 (0.23)	1.16 (0.29)	1.00 (0.22)	1.23 (0.34)
Total lipids in medium LDL, mmol/L	0.52 (0.14)	0.66 (0.18)	0.54 (0.14)	0.69 (0.21)
Total lipids in small LDL, mmol/L	0.34 (0.09)	0.43 (0.11)	0.36 (0.09)	0.45 (0.13)
Total lipids in very large HDL, mmol/L	0.50 (0.19)	0.50 (0.24)	0.61 (0.14)	0.55 (0.23)
Total lipids in large HDL, mmol/L	0.87 (0.28)	0.89 (0.41)	0.79 (0.17)	1.00 (0.35)
Total lipids in medium HDL, mmol/L	0.89 (0.13)	0.96 (0.18)	0.82 (0.09)	1.03 (0.15)
Total lipids in small HDL, mmol/L	1.01 (0.12)	1.08 (0.15)	1.00 (0.07)	1.12 (0.12)
Lipoprotein particle size				
Mean diameter for VLDL particles, nm	37.07 (1.58)	36.97 (1.68)	36.46 (1.29)	35.78 (1.31)
Mean diameter for LDL particles, nm	23.61 (0.11)	23.56 (0.10)	23.64 (0.10)	23.62 (0.11)
Mean diameter for HDL particles, nm	10.10 (0.23)	10.06 (0.28)	10.07 (0.15)	10.10 (0.24)
Cholesterol				
Serum total cholesterol, mmol/L	3.60 (0.63)	4.26 (0.82)	3.94 (0.60)	4.64 (0.86)
Total cholesterol in VLDL, mmol/L	0.45 (0.18)	0.60 (0.28)	0.63 (0.16)	0.67 (0.24)
Remnant cholesterol (non‐HDL, non‐LDL cholesterol), mmol/L	0.97 (0.27)	1.23 (0.38)	1.22 (0.24)	1.38 (0.39)
Total cholesterol in LDL, mmol/L	1.15 (0.34)	1.47 (0.44)	1.23 (0.33)	1.56 (0.50)
Total cholesterol in HDL, mmol/L	1.49 (0.27)	1.56 (0.38)	1.49 (0.21)	1.70 (0.32)
Total cholesterol in HDL2, mmol/L	1.02 (0.25)	1.07 (0.35)	0.89 (0.14)	1.15 (0.29)
Total cholesterol in HDL3, mmol/L	0.47 (0.02)	0.48 (0.03)	0.60 (0.07)	0.55 (0.04)
Esterified cholesterol, mmol/L	2.53 (0.46)	2.99 (0.59)	2.78 (0.43)	3.27 (0.62)
Free cholesterol, mmol/L	1.08 (0.18)	1.27 (0.24)	1.17 (0.18)	1.36 (0.25)
Glycerides and phospholipids				
Serum total triglycerides, mmol/L	1.02 (0.46)	1.28 (0.69)	1.05 (0.38)	1.06 (0.54)
Triglycerides in VLDL, mmol/L	0.70 (0.43)	0.87 (0.63)	0.69 (0.33)	0.64 (0.45)
Triglycerides in LDL, mmol/L	0.118 (0.026)	0.16 (0.04)	0.145 (0.048)	0.17 (0.06)
Triglycerides in HDL, mmol/L	0.132 (0.029)	0.15 (0.04)	0.113 (0.021)	0.138 (0.035)
Total phosphoglycerides, mmol/L	1.63 (0.25)	1.93 (0.34)	1.77 (0.27)	1.94 (0.35)
Ratio of triglycerides to phosphoglycerides	0.54 (0.27)	0.59 (0.32)	0.52 (0.21)	0.46 (0.20)
Phosphatidylcholine and other cholines, mmol/L	1.69 (0.25)	1.98 (0.33)	1.84 (0.27)	2.03 (0.35)
Total cholines, mmol/L	2.00 (0.26)	2.32 (0.35)	2.17 (0.30)	2.35 (0.37)
Apolipoproteins				
Apolipoprotein A1, g/L	1.50 (0.16)	1.59 (0.21)	1.48 (0.11)	1.69 (0.17)
Apolipoprotein B, g/L	0.69 (0.13)	0.82 (0.19)	0.73 (0.12)	0.86 (0.20)
Ratio of apolipoprotein B to apolipoprotein A‐I	0.47 (0.10)	0.52 (0.14)	0.49 (0.09)	0.51 (0.13)
Fatty acids				
Total fatty acids, mmol/L	9.25 (1.65)	10.98 (2.38)	10.24 (1.61)	11.13 (2.17)
Estimated degree of unsaturation	1.21 (0.06)	1.21 (0.06)	1.16 (0.06)	1.21 (0.06)
22:6, docosahexaenoic acid, mmol/L	0.08 (0.03)	0.11 (0.04)	0.11 (0.03)	0.15 (0.05)
18:2, linoleic acid, mmol/L	2.56 (0.46)	2.89 (0.56)	2.83 (0.44)	2.93 (0.54)
Omega‐3 fatty acids, mmol/L	0.30 (0.08)	0.40 (0.12)	0.33 (0.07)	0.44 (0.12)
Omega‐6 fatty acids, mmol/L	3.08 (0.49)	3.52 (0.61)	3.43 (0.49)	3.71 (0.63)
Polyunsat. fatty acids, mmol/L	3.38 (0.55)	3.93 (0.70)	3.76 (0.54)	4.15 (0.70)
Monounsat. fatty acids; 16:1, 18:1, mmol/L	2.52 (0.62)	3.09 (0.93)	2.39 (0.57)	2.82 (0.80)
Saturated fatty acids, mmol/L	3.35 (0.64)	3.96 (0.93)	4.08 (0.67)	4.16 (0.82)
Fatty acid ratios				
Ratio of 22:6 docosahexaenoic acid to total fatty acids (%)	0.83 (0.25)	1.03 (0.28)	1.07 (0.22)	1.32 (0.35)
Ratio of 18:2 linoleic acid to total fatty acids (%)	27.81 (3.24)	26.66 (3.43)	27.75 (2.41)	26.48 (2.80)
Ratio of omega‐3 fatty acids to total fatty acids (%)	3.27 (0.59)	3.70 (0.71)	3.25 (0.52)	3.97 (0.85)
Ratio of omega‐6 fatty acids to total fatty acids (%)	33.55 (3.11)	32.48 (3.37)	33.68 (2.62)	33.56 (2.64)
Ratio of polyunsat. fatty acids to total fatty acids (%)	36.82 (3.35)	36.18 (3.62)	36.94 (2.75)	37.53 (2.83)
Ratio of monounsat. fatty acids to total fatty acids (%)	26.99 (2.61)	27.81 (2.90)	23.21 (2.72)	25.07 (2.88)
Ratio of saturated fatty acids to total fatty acids (%)	36.19 (1.74)	36.00 (1.96)	39.86 (1.77)	37.39 (1.56)
Glycolysis related				
Glucose, mmol/L	3.86 (0.64)	3.88 (0.85)	4.18 (0.52)	4.48 (0.88)
Lactate, mmol/L	1.73 (0.46)	1.56 (0.46)	1.36 (0.48)	0.80 (0.34)
Citrate, mmol/L	0.13 (0.02)	0.11 (0.02)	0.13 (0.03)	0.094 (0.03)
Amino acids				
Alanine, mmol/L	0.39 (0.06)	0.40 (0.06)	0.29 (0.06)	0.25 (0.06)
Glutamine, mmol/L	0.48 (0.05)	0.46 (0.07)	0.54 (0.06)	0.47 (0.06)
Histidine, mmol/L	0.07 (0.01)	0.07 (0.01)	0.08 (0.01)	0.06 (0.01)
Isoleucine, mmol/L	0.05 (0.02)	0.06 (0.02)	0.05 (0.02)	0.03 (0.01)
Leucine, mmol/L	0.07 (0.02)	0.08 (0.02)	0.06 (0.02)	0.05 (0.01)
Valine, mmol/L	0.16 (0.04)	0.17 (0.04)	0.14 (0.04)	0.14 (0.03)
Phenylalanine, mmol/L	0.07 (0.01)	0.07 (0.01)	0.04 (0.01)	0.04 (0.01)
Tyrosine, mmol/L	0.05 (0.01)	0.05 (0.01)	0.07 (0.02)	0.05 (0.01)
Ketone bodies				
Acetate, mmol/L	0.031 (0.005)	0.04 (0.04)	0.058 (0.024)	0.064 (0.028)
Acetoacetate, mmol/L	0.031 (0.018)	0.03 (0.02)	0.053 (0.048)	0.034 (0.026)
3hydroxybutyrate, mmol/L	0.12 (0.09)	0.112 (0.084)	0.07 (0.10)	0.11 (0.12)
Fluid balance				
Creatinine, mmol/L	0.040 (0.007)	0.06 (0.01)	0.036 (0.006)	0.062 (0.010)
Albumin (signal area)	0.093 (0.005)	0.09 (0.01)	0.090 (0.004)	0.091 (0.004)

ex.large indicates extra large; HDL, high‐density lipoprotein; IDL, intermediate‐density lipoprotein; LDL, low‐density lipoprotein; polyunsat., polyunsaturated; VLDL, very‐low‐density lipoprotein.

### Metabolomic Measures and cIMT

**Figure 3:**
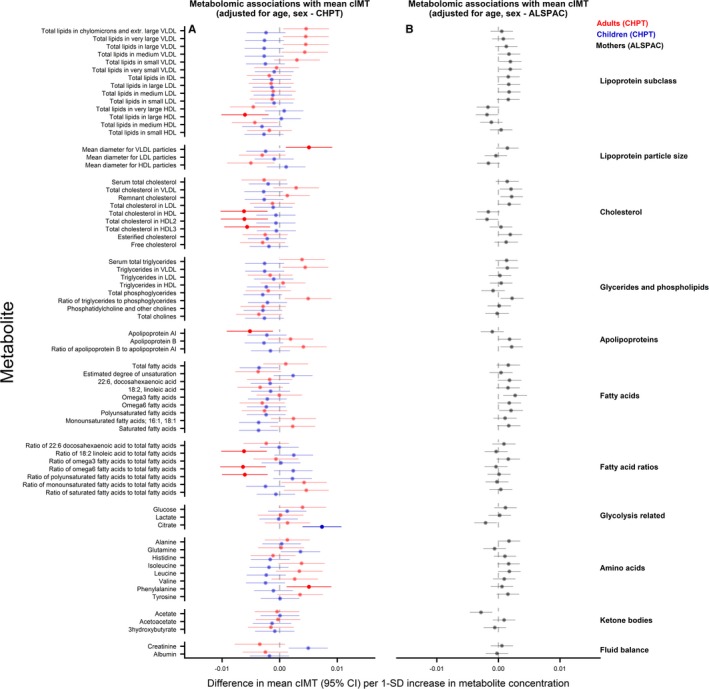
Associations between metabolomic measures and carotid intima‐media thickness (cIMT) in children and adults. Association measures are difference in mean cIMT per 1‐SD unit increase in metabolite for **A**) CheckPoint children (blue), CheckPoint adults (red), and **B**) The Avon Longitudinal Study of Parents and Children (ALSPAC) mothers (gray) adjusted for age and sex. Error bars represent 95% confidence intervals (CI). Significant associations after *P* values adjusted for multiple testing using the Benjamini–Hochberg procedure are shown in bold (false discovery rate=0.10). CHPT indicates Child Health CheckPoint; HDL, high‐density lipoprotein; IDL, intermediate‐density lipoprotein; LDL, low‐density lipoprotein; VLDL, very‐low‐density lipoprotein.

**Figure 4:**
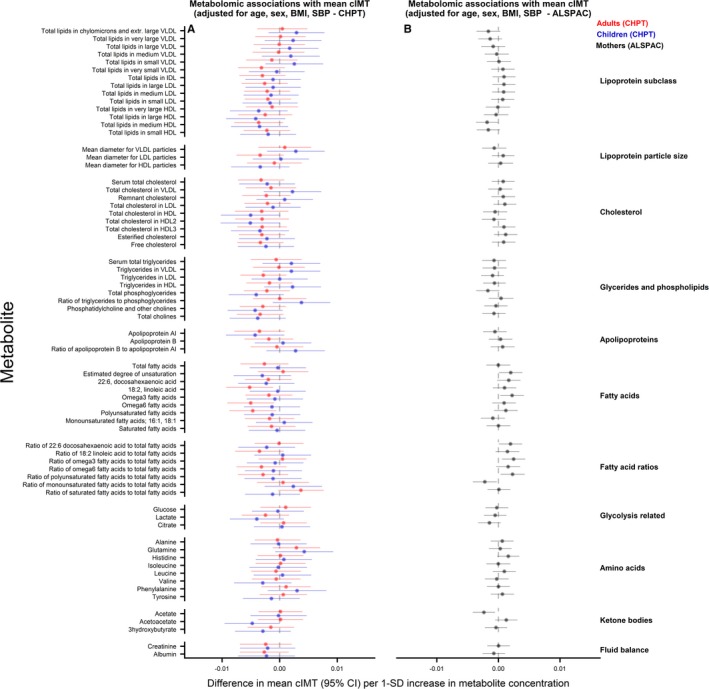
Associations between metabolomic measures and carotid intima‐media thickness (cIMT) in children and adults. Association measures are difference in mean cIMT per 1‐SD unit increase in metabolite for **A**) CheckPoint children (blue), CheckPoint adults (red), and **B**) The Avon Longitudinal Study of Parents and Children (ALSPAC) mothers (gray) adjusted for age, sex, body mass index, and blood pressure. Error bars represent 95% confidence intervals (CI). Significant associations after *P* values adjusted for multiple testing using the Benjamini–Hochberg procedure are shown in bold (false discovery rate=0.10). CHPT indicates Child Health CheckPoint; HDL, high‐density lipoprotein; IDL, intermediate‐density lipoprotein; LDL, low‐density lipoprotein; VLDL, very‐low‐density lipoprotein.

There was no replication of any statistically significant associations across ages or cohorts. In age‐ and sex‐adjusted analyses, citrate was positively associated with cIMT in CheckPoint children, mean diameter of very‐low‐density lipoprotein–related particles and phenylalanine were positively and HDL‐related measures, apolipoprotein A‐1, and fatty acid ratios inversely associated with cIMT in CheckPoint adults (Figure 3). In ALSPAC adults, there was no evidence of an association between any metabolite measure and cIMT. In both ages and both cohorts, associations of metabolic measures with cIMT attenuated toward null following additional adjustment for BMI and systolic BP (Figure 4).

### Metabolomic Measures and PWV

**Figure 5:**
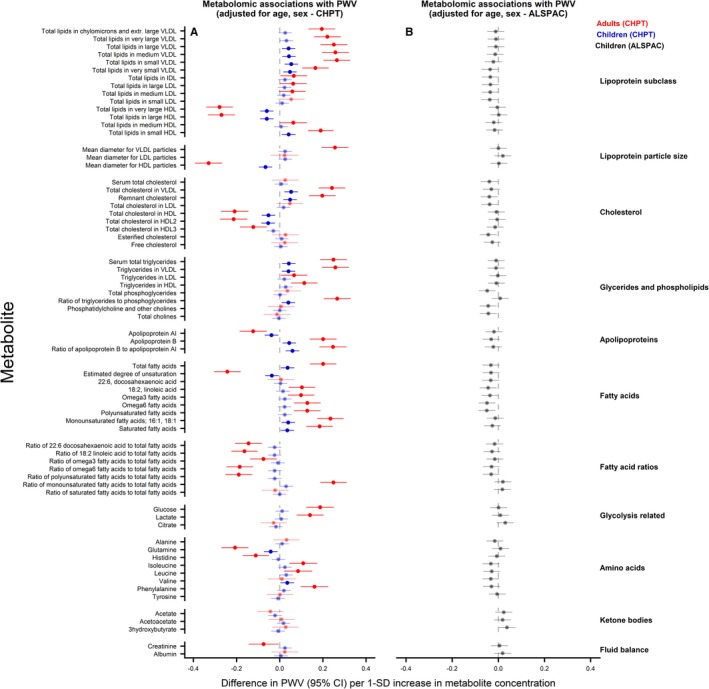
Associations between metabolomic measures and pulse wave velocity (PWV) in children and adults. Association measures are difference in PWV per 1‐SD unit increase in metabolite concentration for **A**) CheckPoint children (blue), CheckPoint adults (red), and **B**) The Avon Longitudinal Study of Parents and Children (ALSPAC) children (gray) adjusted for age and sex. Error bars represent 95% confidence intervals (CI). Significant associations after *P* values adjusted for multiple testing using the Benjamini–Hochberg procedure are shown in bold (false discovery rate=0.10). CHPT indicates Child Health CheckPoint; HDL, high‐density lipoprotein; IDL, intermediate‐density lipoprotein; LDL, low‐density lipoprotein; VLDL, very‐low‐density lipoprotein.

**Figure 6:**
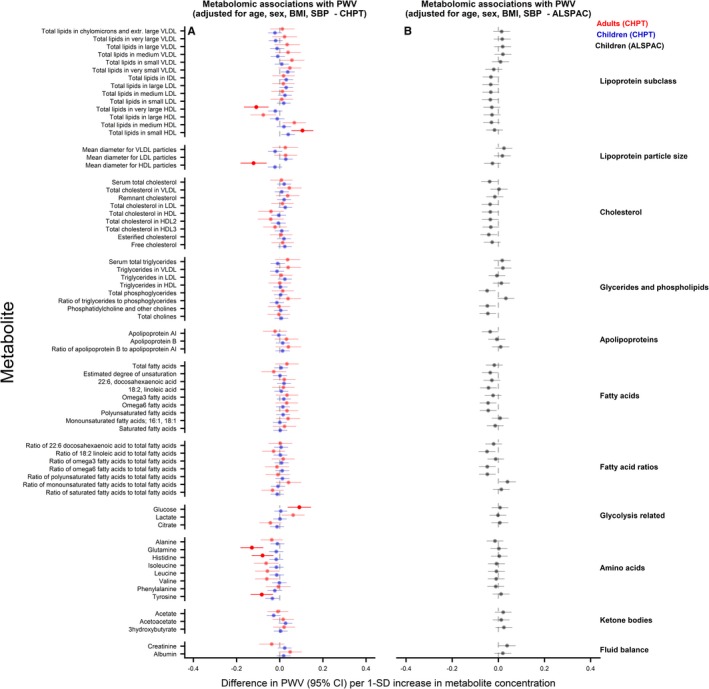
Associations between metabolomic measures and pulse wave velocity (PWV) in children and adults. Association measures are difference in PWV per 1‐SD unit increase in metabolite concentration for **A**) CheckPoint children (blue), CheckPoint adults (red), and **B**) The Avon Longitudinal Study of Parents and Children (ALSPAC) children (gray) adjusted for age, sex, body mass index, and blood pressure. Error bars represent 95% confidence intervals (CI). Significant associations after *P* values adjusted for multiple testing using the Benjamini–Hochberg procedure are shown in bold (false discovery rate=0.10). CHPT indicates Child Health CheckPoint; HDL, high‐density lipoprotein; IDL, intermediate‐density lipoprotein; LDL, low‐density lipoprotein; VLDL, very‐low‐density lipoprotein.

Results from age‐ and sex‐adjusted analyses are shown in Figure 5. Very‐low‐density lipoprotein– and triglyceride‐derived measures, apolipoprotein B, and several fatty acids were positively and HDL‐related measures, apolipoprotein A‐I, and glutamine inversely associated with PWV in CheckPoint children and adults. Valine was positively associated with PWV in CheckPoint children, whereas glucose, lactate, isoleucine, leucine, and phenylalanine were positively and histidine and creatinine inversely related with PWV in CheckPoint adults. In ALSPAC children, there was no evidence of an association between any metabolite and PWV. With further adjustment for BMI and systolic BP (Figure 6), all associations attenuated to the null in CheckPoint children. In CheckPoint adults, glucose and the amount of total lipids in small HDL were positively and total lipids in very large HDL, mean diameter of HDL, glutamine, histidine, and tyrosine were inversely associated with PWV.

## Discussion

Our analyses of 2 large, independent, population‐based studies revealed limited evidence of an association between NMR‐based metabolomic profile and subclinical phenotypes of atherosclerosis in children following adjustment for age and sex. These associations were generally attenuated toward the null with further adjustment for BMI and systolic BP. In the CheckPoint adults, but not CheckPoint or ALSPAC children, glucose was positively and some HDL‐cholesterol derived measures and amino acids (glutamine, histidine, and tyrosine) were inversely associated with PWV independently of BMI and systolic BP.

We were unable to identify other published studies of the associations of multiple metabolites with cIMT or PWV in children. Several studies in adults have shown associations with cardiovascular events. For example, a previous study of 3 independent adult cohorts from Finland and the UK (total N=13 441) found robust (replicated), prospective associations between NMR‐derived measures (using the same NMR platform as here) and cardiovascular events, even with adjustment for conventional risk factors (age, sex, lipids, BP, smoking, diabetes mellitus, and medication).^27^ Specifically, phenylalanine and monounsaturated fatty acids were positively associated with subsequent CVD event risk, whereas omega‐6 fatty acids and docosahexaenoic acid levels were inversely associated.^27^ Furthermore, a systematic review of prospective studies reported evidence of associations of multiple metabolites with CVD.^8^ Given these existing findings and evidence that the arterial antecedents of CVD begin in childhood, we hypothesized that multiple metabolites would be associated with measures of arterial disease in children. However, we found no evidence for this in our cross‐sectional and relatively short (3‐year follow‐up) longitudinal analyses. This may indicate that a longer exposure period to adverse metabolite levels is needed to observe an association with the subclinical vascular phenotypes. It is also important to consider that PWV and cIMT are surrogate measures of atherosclerosis, whereas the occurrence of cardiovascular events requires both an underlying atherosclerotic plaque formation and different factors that lead to plaque instability and rupture.

In contrast to studies of adults both younger and older than our study population, we found little evidence of associations between metabolite measures and cIMT in adults. Specifically, a study of 1297 participants (mean age, 60 years), focusing on amino acids, found that circulating tyrosine, phenylalanine, and isoleucine were associated with cIMT,^28^ whereas a second NMR‐based prospective study (N=1595; mean age at baseline, 32 years) linked docosahexaenoic acid, tyrosine, and glutamine with cIMT progression.^29^ Replication was not undertaken for either study, but in both reports, tyrosine was related to subclinical atherosclerosis. For the lipid measures, in an earlier study utilizing a similar NMR‐based methodology, several HDL‐cholesterol subgroups and HDL particle size have been shown to predict future lower risk of cardiovascular events.^27^ Similar findings have been reported on the association with cIMT^30^ and 6‐year intima‐media thickness progression.^29^ Of those factors previously associated with cIMT, we observed that tyrosine and glutamine were associated with PWV among CheckPoint adults (no PWV data were available in ALSPAC adults). However, unlike previous associations with cIMT (ie, higher level of metabolite related with thicker/worse carotid artery structure),^28^, ^29^ we found tyrosine and glutamine to be inversely associated with PWV (ie, higher level of metabolite was associated with better arterial function). The inverse association with glutamine is consistent with the previous meta‐analysis of 3 independent cohorts described above, which found an inverse association with incident CVD.^27^ In keeping with earlier studies on cIMT, we observed that HDL‐cholesterol–related measures were inversely associated with PWV among CheckPoint adults. In previous studies of the associations between metabolites and PWV, Menni et al^31^ observed, among 1797 female twins (mean age, 58 years), that 12 metabolites, including glutamine, were inversely associated with PWV in BMI‐adjusted models. In a case‐control study among males with/without peripheral artery disease, tyrosine levels were positively associated with PWV in the case group.^32^

There is increasing interest in the utility of the metabolomic profile as a part of clinical risk prediction, particularly in the primary prevention of CVD. Our findings from general populations do not support the introduction of NMR metabolic profiles into pediatric cardiovascular practice. Among adults, we observed some signals related to preclinical atherosclerosis, but the most robust evidence to date with this NMR platform comes from prospective analyses with disease end points and replication across 3 independent cohorts.^27^

To our knowledge, this is the first study to examine the metabolomics profile of cardiovascular phenotypes among children. Importantly, we analyzed data from 2 independent cohorts that used the same NMR metabolomics platform. Some limitations to the present study warrant consideration, particularly the cross‐sectional nature of most of the data, which limits causal inference. The childhood analyses between CheckPoint and ALSPAC cohorts are not entirely comparable, given that CheckPoint analyses were cross‐sectional, whereas in the ALSPAC they were prospective. The lack of any associations in the ALSPAC, even in age‐ and sex‐ adjusted analyses, might imply that the metabolites analyzed are not causally related to subclinical atherosclerosis in children, but further prospective analyses are warranted. Neither study had data on the progression of atherosclerotic phenotypes. The ALSPAC study did not have data on PWV in adults, nor cIMT in children. In addition, PWV measures differed between the 2 cohorts (carotid‐femoral PWV in CheckPoint, carotid‐radial PWV in the ALSPAC). The adult participants in both cohorts were not representative of the general populations given that they largely comprised mothers. We have previously shown that the CheckPoint cohort is under‐represented of socioeconomically disadvantaged families,^22^ and although ALSPAC reflects the sociodeomographic characteristics of the local (South West of England) population from which it was drawn, it is more affluent and less ethnically diverse than the rest of the UK. Thus, results from the adults in our study may not generalize to men, and in both age groups may not generalize to non‐white European ethnicities or those from more‐deprived socioeconomic backgrounds.

In conclusion, the present data suggest that, among children, blood metabolites have no independent association with cIMT and arterial PWV after adjustment for BMI and BP. In adults, these data suggest that glucose levels are positively and some HDL‐related measures and amino acids inversely associated with lower PWV, but this requires further replication.

## Sources of Funding

The CheckPoint has been supported to date by the National Health and Medical Research Council of Australia (1041352, 1109355), The Royal Children's Hospital Foundation (2014‐241), Murdoch Children's Research Institute, The University of Melbourne, National Heart Foundation of Australia (100660), the Australian Department of Social Services, and the Financial Markets Foundation for Children (2014‐055: 2016‐310).

The UK Medical Research Council and the Wellcome Trust (Grant ref: 102215/2/13/2) and the University of Bristol provide core support for ALSPAC. The work presented here has been supported by the European Research Council under the European Union's Seventh Framework Programme (FP7/2007‐2013)/ERC grant agreement 669545 (DevelopObese), the National Institutes of Health (R01 DK10324), and the European Union's Horizon 2020 Research and Innovation Programme under grant agreement 733206 (LifeCycle). Lawlor and Santos Ferreira work in a unit that receives funding from the UK Medical Research Council, and Lawlor is a UK National Institute of Health Research Senior Investigator (MC_UU_00011/6). Lawlor is a National Institute of Health Research Senior Investigator (NF‐SI‐0166‐10196).

The following authors were supported by the National Health and Medical Research Council of Australia: Senior Research Fellowships (1046518) to Wake and (1064629) Burgner. Juonala is supported by the Federal Research Grant of Finland to Turku University Hospital, Finnish Cardiovascular Foundation, Juho Vainio Foundation, and the Murdoch Children's Research Institute (Dame Elizabeth Murdoch Fellowship). Research at the Murdoch Children's Research Institute is supported by the Victorian Government's Operational Infrastructure Program. The Heart Research Group at Murdoch Children's Research Institute is supported by the Royal Children's Hospital 1000, Royal Children's Hospital Foundation, and Big W. The funding bodies did not play any role in the study. The views expressed in this article are those of the authors and not necessarily any funders or anyone acknowledged.

## Disclosures

Lawlor, in addition to national and international government and charity funding, has received support from Medtronic Ltd and Roche Diagnostics for work unrelated to this article. The remaining authors have no disclosures to report.
